# Nasopharyngeal *Staphylococcus aureus* colonization among HIV-infected children in Addis Ababa, Ethiopia: antimicrobial susceptibility pattern and association with *Streptococcus pneumoniae* colonization

**DOI:** 10.1099/acmi.0.000557.v3

**Published:** 2023-08-16

**Authors:** Henok Alemu Gebre, Ashenafi Alemu Wami, Eyerusalem Solomon Kebede, Melaku Yidnekachew, Meseret Gebre, Abel Abera Negash

**Affiliations:** ^1^​ Armauer Hansen Research Institute (AHRI), Addis Ababa, Ethiopia; ^2^​ Department of Microbiology, Immunology and Parasitology, School of Medicine, Addis Ababa University, Addis Ababa, Ethiopia

**Keywords:** antimicrobial susceptibility, Ethiopia, HIV-infected, MRSA, nasopharyngeal colonization, pediatrics, *Staphylococcus aureus*, *Streptococcus pneumoniae*

## Abstract

**Background.:**

*
Staphylococcus aureus* and *Streptococcus pneumoniae* are common inhabitants of the nasopharynx of children. HIV-infected children have higher risk of invasive diseases caused by these pathogens. With widespread use of pneumococcal conjugate vaccines and the emergence of methicillin-resistant *S. aureus *, the interaction between S. *aureus* and *S. pneumoniae* is of a particular significance. We sought to determine the magnitude of colonization by methicillin-sensitive and -resistant *S. aureus* and colonization by *S. pneumoniae *; associated risk factors and antimicrobial susceptibility pattern among HIV-infected children in Addis Ababa, Ethiopia.

**Method.:**

A prospective observational study was conducted among 183 HIV-infected children at ALERT hospital Addis Ababa, Ethiopia from September 2016 to August 2018. *S. aureus* and *S. pneumoniae* were identified using standard bacteriological techniques, antimicrobial susceptibility testing was performed on *S. aureus* and screening for methicillin resistance was carried out by amplifying the *mecA* gene. Risk factors were analysed by using binary logistic regression.

**Results.:**

The prevalence of nasopharyngeal *S. aureus *, MRSA and *S. pneumoniae* colonization were 27.3, 2.7 and 43.2 %, respectively. Multivariable analysis indicated an inverse association between *S. aureus* and *S. pneumoniae* nasopharyngeal colonization [aOR, 0.49; CI, (0.24, 0.99); *P=0.046*]. The highest level of resistance in both methicillin-sensitive *S. aureus* (MSSA) and MRSA was observed against tetracycline.

**Conclusions:**

. We found an inverse association between *S. aureus* and *S. pneumoniae* colonization among HIV-infected children. Continued assessment of the impact of pneumococcal conjugate vaccines and antiretroviral therapy on nasopharyngeal bacterial ecology is warranted.

## Data Summary

All the data used in the study is available within the manuscript.

## Introduction

*Staphylococcus aureus* is a human commensal and also causes clinically important community and hospital-acquired infections such as endocarditis, bacteremia, osteomyelitis and skin and soft tissue infections [1]. *S. aureus* colonizes multiple body sites in humans including anterior nares, skin, perineum and pharynx. However, the most frequent carriage site for *S. aureus *, with a 27 % carriage rate among adults, is the nose and a causal relationship exists between *S. aureus* colonization and infection [2]. Compared to adults, persistent carriage is mostly seen in children and carriage rates vary between 45 % in the first 8 weeks and decline to 21 % by 6 months of age [3].

Methicillin-resistant *Staphylococcus aureus* (MRSA) was discovered in 1961, soon after the introduction of methicillin in clinical isolates and then in the 1980s in the community. It has since spread globally and its prevalence is increasing resulting in increased health-care costs, morbidity and mortality [1, 4]. HIV-infected patients, due to their weakened immune system, are at increased risk for several infections, including those caused by *S. aureus* and MRSA [5]. The morbidity and mortality associated with MRSA in patients with HIV infection [6] makes the study of MRSA colonization among the HIV-infected patients of particular interest. In addition, colonization with MRSA has been associated with a fourfold increase in the risk of infection [7].

Studies indicate that compared to healthy individuals, the prevalence of MRSA nasal colonization is higher in HIV-positive people [8, 9]. The risk of MRSA colonization often mirrors that of infection and additional people with higher risk of colonization/infection are, children, those in prisons, military recruits, those in poor neighbourhoods, livestock workers, individuals with prior MRSA infection and hospitalization and those with cystic fibrosis [1]. The main risk factors for colonization and infection among HIV-infected patients on the other hand are use of antimicrobials, previous hospitalization and low CD4 +T lymphocyte counts [10].

MRSA is now considered an urgent threat to public health and among the priority list of pathogens for which new antibiotics are required [11]. In order to make effective treatment of patients with disease due to MRSA and to prevent further transmission, accurate detection of methicillin resistance is of the utmost importance [12].

The *mecA* gene is highly conserved in staphylococcal strains and thus is a useful marker and is considered as the gold standard for identifying methicillin resistance in *S. aureus* isolates. The *mecA* gene is located on the staphylococcal chromosome cassette *mec* and encodes penicillin binding protein 2 a (PBP2a) [12]. Other chromosomal factors such as *femA* and *femB* are also associated with the expression of methicillin resistance [13].

Pneumococcal conjugate vaccines (PCVs) began being introduced in infant immunization schedules from the year 2000 and studies indicate that administration of PCVs to infants modifies not only the carriage of *Streptococcus pneumoniae* but also that of *S. aureus* and there is an inverse relationship between the carriage of the two bacteria [14, 15]. There was also an earlier clinical trial that indicated an increase in acute otitis media due to *S. aureus* after PCV vaccination [16]. We have recently been able to show that 5–6 years after introduction of PCV10 in Ethiopia, *S. aureus* was the main cause of bacteremic community-acquired pneumonia among children [17].

Earlier studies in HIV-infected children indicated a lack of association between *S. pneumoniae* and *S. aureus* carriage and cite suboptimal adaptive immunity as a possible reason [14] but there are suggestions that *S. pneumoniae – S. aureus* interference is CD4^+^ T cell-mediated and returns to normal following antiretroviral therapy (ART) [18].

In Ethiopia, PCV10 was introduced in October 2011 as a three-dose primary series (3*P*+0) without any booster dose and a decision has now been made to switch to PCV13. A previous study among HIV-infected children in Northern Ethiopia indicated a pharngeal carriage prevalence of 29 % for *S. aureus* and 12.3 % for *S. pneumoniae* [19]. There is however a scarcity of data in Ethiopia on the relationship between the nasopharyngeal carriage of *S. pneumoniae* and *S. aureus* and the possible impact of PCV introduction especially among HIV-infected children. The aim of this study was therefore to determine the magnitude of methicillin-sensitive and -resistant *S. aureus* nasopharyngeal colonization and their association with *S. pneumoniae* colonization among HIV-infected children in Addis Ababa, Ethiopia.

## Methods

### Study design and setting

A prospective observational study was conducted at the All Africa Leprosy Rehabilitation and Training Hospital (ALERT), a governmental referral hospital, located in Addis Ababa, Ethiopia. The target population of this study was HIV-positive children aged 0–15 years coming for ART follow-up to the ALERT paediatric HIV clinic from 1 September 2016 to 31 August 2018.

### Patient enrollment and data collection

Trained research nurses approached the parents or guardians of the HIV-infected children who came for an initiation or followup visit at the ART clinic and briefed them on the information that has been provided in the patient information sheet. Children of parents who gave consent were then included in the study. Study participants' demographic and clinical data including information on risk factors for colonization and disease were obtained and transferred to the questionnaire prepared for the study.

The dependent variable assessed was colonization with *S. aureus *. The independent variables assessed were gender, age, number of household members, number of rooms in the house, presence of siblings <5 years of age, parents' level of education, monthly income, exposure to cigarette smoke, reasons for hospital visit, antiretroviral treatment status, PCV vaccination, *S. pneumoniae* colonization, upper respiratory tract infection, lower respiratory tract infection within the last 3 months, CD4^+^ T cell count, and previous hospitalization within the last 3 months.

### Specimen collection

Nasopharyngeal (NP) swabs were collected from 183 HIV-infected consecutive children using flocked mini tip rayon swabs (Copan, Brescia, Italy). Briefly, tipping the head of the children slightly backward, the swab was directly inserted parallel to the base of the NP passage until reaching the nasopharynx, which is located about one-half to two-thirds the distance from the nostril to the ear lobe. Once the swab was fully passed into the nasopharynx, it was rotated 180^o^ and left in place for 5 s to saturate the swab tip. The swab was then removed slowly and immediately transferred into cryovials containing 1 ml of STGG (skim-milk, tryptone, glucose, and glycerol) transport medium. The inoculated STGG was initially stored at −20 °C in the hospital ward. Samples were then transported to the Armauer Hansen Research Institute (AHRI) laboratory using ice and were frozen at −80 ˚C until further analysis.

### Culture and identification

The samples were initially thawed and 100 -µl of the NP swab sample was plated on a blood agar plate (BAP) with and without gentamicin by streaking the sample using a sterile loop. BAP plates were then incubated at 37 °C with ~5 % CO_2_ for 18–24 h. *S. aureus* was identified using colony morphology on sheep blood agar, gram staining, catalase activity, production of coagulase and growth on mannitol salt agar. Identification of *S. pneumoniae* was made on the basis of colony morphology, optochin susceptibility and bile solubility.

### Antimicrobial susceptibility testing

The susceptibility of *S. aureus* isolates to chloramphenicol (30 µg), ciprofloxacin (5 µg), clindamycin (2 µg), erythromycin (15 µg), gentamicin (10 µg), tetracycline (30 µg) and trimethoprim/sulfamethoxazole (1.25 µg/23.75 µg) was determined by the disc diffusion method on Mueller–Hinton agar (Oxoid, Basingstoke, UK). Test results were interpreted according to the Clinical and Laboratory Standard Institute criteria. Multidrug resistance (MDR) was defined as resistance to three or more antimicrobial classes. *S. aureus* (ATCC 25923) was used as a quality control organism.

### Detection of mecA genes

DNA extraction was performed by boiling *S. aureus* pellets in 300 µl of TE buffer as described previously [20]. Detection of 310 bp fragment of *mecA* was performed using primer pairs: 5′-GTAGAAATGACTGAACGTCCGATAA-3′ and

5′ CCAATTCCACATTGTTTCGGTCTAA −3′ as described previously [21]. The reaction mixture contained 12.5 µl of hot star master mix (Qiagen, Hilden, Germany), 0.5 µl each of the forward and reverse primers, 9 µl of molecular grade water and 2.5 µl of the template with a final volume of 25 µl. Amplification was carried out with 40 cycles of initial heat activation at 95 °C for 15 min, denaturation at 94 °C for 30 s, followed by annealing at 52 °C for 45 s, extension at 72^ °^C for 1 min, and final extension at 72^ °^C for 10 min. The PCR products were analysed by electrophoresis on a 2 % agarose gel.

### Statistical analysis

Data were initially entered in ReDCap (Vanderbilt University, Nashville, TN, USA), exported into Excel and analysed using PASW Statistics 20 software (SPSS, Chicago, IL, USA). Sociodemographic, environmental and clinical characteristics were analysed using descriptive statistics. Bivariate analysis, using binary logistic regression, was initially performed in order to determine factors associated with *S. aureus* colonization. Multivariate logistic regression was then used to assess independent associations for variables significant at *P*< 0.1. Variables at *P*<0.05 were then considered statistically significant in the multivariable analysis.

### Ethical considerations

The study procedures were in accordance with the Helsinki Declaration. The study protocol was approved by the AHRI/ALERT Ethical Review Committee (AAERC) (PO/017/2015). An 0fficial permission letter was obtained from the ALERT hospital. A written informed assent and consent was obtained from study participants, and parents or guardians of children, respectively, before including them in the study. The study participants’ right to refuse or not give nasopharyngeal samples without affecting their routine medical services was granted. Samples were coded to keep the confidentiality of the study participants' personal information.

## Results

### Socio-demographic and clinical characteristics of the study participants

Out of the potentially eligible 750 HIV-infected children, nasopharyngeal samples were collected from a total of 183 HIV-infected children at the ALERT hospital. Among the study participants, 50.8 %(93/183) were girls; the mean age of the study participants was 10.78±2.68 years. The median [interquartile range (IQR)] CD4 +T cell count of the study participants was 73(524-924) cells/mm^3^ and 95.6 %(175/183) of the children have received full dose of PCV10 (Table 1).

**Table 1.** Socio-demographic, environmental and clinical characteristics and their association with *S. aureus* colonization among HIV-infected children aged <15 years at ALERT hospital, Addis Ababa, Ethiopia

Variable	Category	All children (*n*=183)	Colonized with * S. aureus * (*n*=50)	Bivariate analysis	
		No. (%)	No. (%)	Cor (95 % CI)	*P*§
**Gender**	Male	90 (49.2)	25 (50)	1.05 (0.55–2.01)	0.892
	Female	93 (50.8)	25 (50)	Ref	
**Age category (in years)**	0–4	3 (1.6)	2 (4)	5.18 (0.45–58.75)	1.86
	5–9	51 (27.8)	12 (24)	0.8 (0.37,1.69)	.550
	10–14	129 (70.4)	36 (72)	Ref	
**Number of household members**	<=3	37 (20.2)	11 (22)	0.65 (0.24–1.76)	.396
	4–6	113 (61.7)	26 (52)	0.41 (0.17–0.97)	.065
	>=7	33 (18)	13 (26)	Ref	
**Number of rooms in the house**	1	69 (37.7)	16 (32)	0.71 (0.36–1.41)	0.330
	>=2	114 (62.3)	34 (68)	Ref	
**Siblings aged <5 years**	Yes	68 (37.2)	13 (26)	0.5 (0.24–1.03)	0.58
	No	115 (62.8)	37 (74)	Ref	
**Mother's level of education**	Illiterate	68 (37.2)	22 (44)	2.1 (0.18–24.59)	0.555
	First level	72 (39.3)	18 (36)	1.25(0.12–13)	0.852
	Secondary	39 (21.3)	9 (18)	1 (0.09–10.23)	1.000
	College and above	4 (2.2)	1 (2)	Ref	
**Father's level of education**	Illiterate	60 (32.8)	19 (38)	1.1 (0.26–5.03)	0.860
	First level	58 (31.7)	13 (26)	0.82 (0.2–3.27)	0.773
	Secondary	53 (29)	14 (28)	0.58 (0.15–2.23)	0.426
	College and above	12 (6.6)	4 (8)	Ref	
**Monthly income (in Ethiopian Birr)**	<1000	108 (59)	31 (62)	1.61 (0.43–6.10)	0.483
	1000–2500	43 (23.5)	11 (22)	1.38 (0.33–5.8)	0.664
	2500–3500	17(9.3)	5(10)	1.67(0.32–8.6)	0.541
	>3500	15 (8.2)	3 (6)	Ref	
**Exposure to cigarette smoking**	Yes	7 (3.8)	1 (2)	0.37 (0.04–3.1)	0.355
	No	176 (96.2)	49 (98)	Ref	
**Reason for hospital visit**	ART follow-up	85 (46.6)	26 (52)	1.1 (02–6.05)	0.911
	Asthma	1 (.5)	1 (2)	–	1.000
	Dermatological problems	18 (9.8)	7 (14)	1.59 (0.24–10.56)	0.63
	LRTI symptoms	72 (39.3)	14 (28)	0.6 (0.11–3.44)	0.570
	UTI	7 (3.8)	2 (4)	Ref	
**Antiretroviral treatment status**	Follow-up	149 (81.4)	41 (82)	0.9 (0.39–2.09)	0.812
	Initiation	34 (18.6)	9 (18)	Ref	
**PCV10 vaccine status**	Full dose†	175 (95.6)	45 (90)	3.58 (0.92–13.93)	0.065
	Incomplete‡	8 (4.4)	5 (10)	Ref	
*** S. pneumoniae* carriage**	Yes	79 (43.2)	15 (30)	0.46 (0.23–0.93)	**0.029**
	No	104 (56.8)	35 (70)	Ref	
**URTI in the last 3** **months**	Yes	25 (13.7)	8 (16)	1.81 (0.61–5.4)	0.495
	No	158 (86.3)	42 (84)	Ref	
**LRTI in the last 3** **months**	Yes	6 (3.3)	2 (4)	1.34 (0.24–7.58)	0.738
	No	177 (96.7)	48 (96)	Ref	
**CD4^+^ T cell count** **(cells/mm^3^)**	>350	164 (89.6)	46 (92)	0.73 (0.23–2.34)	0.600
	<350	19 (10.4)	4 (8)	Ref	
**Previous hospitalization in the last 3** **months**	Yes	6 (3.3)	47 (94)	2.77 (0.54–14.18)	0.223
	No	177 (96.7)	3 (6)	Ref	

*LRTI symptoms represent (cough, difficulty in breathing and runny nose).

†Full dose PCV10 indicates all three doses.

‡Incomplete PCV10 vaccination includes ≤ 2 doses.

§*P* value of binary logistic regression analysis, Values in bold: *P* < 0.05.

CAP, community-acquired pneumonia; CI, 95% confidence interval; COR, Crude odds ratio; LRTI, lower respiratory tract infection; PCV, pneumococcal conjugate vaccine; URTI, upper respiratory tract infection; UTI, urinary tract infection.

### Prevalence of *S. aureus* and *S. pneumoniae* colonization

The overall prevalence of *S. aureus* nasopharyngeal colonization among the HIV-infected children in this study was 27.3 %(50/183) whereas. *S. pneumoniae* was isolated from 43.2 %(79/183) of the children (Table 1).

### Prevalence of MRSA colonization and detection of *mecA*

*mecA* was detected in 10 %(5/50) of the *S. aureus* isolates and MRSA colonization rate was therefore 2.7 %(5/183).

### Risk factors associated with *S. aureus* colonization

According to multivariable logistic regression analysis, there was a statistically significant association between *S. aureus* and *S. pneumoniae* nasopharyngeal colonization [aOR, 0.49; CI, (0.24–0.99); *P=0.046*] (Table 2).

**Table 2.** Risk factors associated with *S. aureus* colonization among HIV-infected children aged <15 years at ALERT hospital, Addis Ababa, Ethiopia

	Bivariate		Multivariable	
**Variable**	**cOR (Cl)**	P*	**aOR (CI)**	P*
PCV10 vaccine status	3.58 (0.92–13.93)	0.065	2.81 (0.7–11.34)	0.148
* S. pneumoniae* carriage	0.46 (0.23–0.93)	**0.029**	0.49 (0.24–0.99)	**0.046**
Number of household members (4–6)	0.46 (0.2–1.05)	0.065	0.49 (0.21, 1.15)	0.104

^∗^ *P* value of binary logistic regression analysis. Values in bold: *P*<0.05.

aOR, adjusted odds ratio; CI, 95% confidence interval; COR, Crude odds ratio; PCV, pneumococcal conjugate vaccine.

### Antibiotic susceptibility pattern of *S. aureus* isolates

**Fig. 1.**
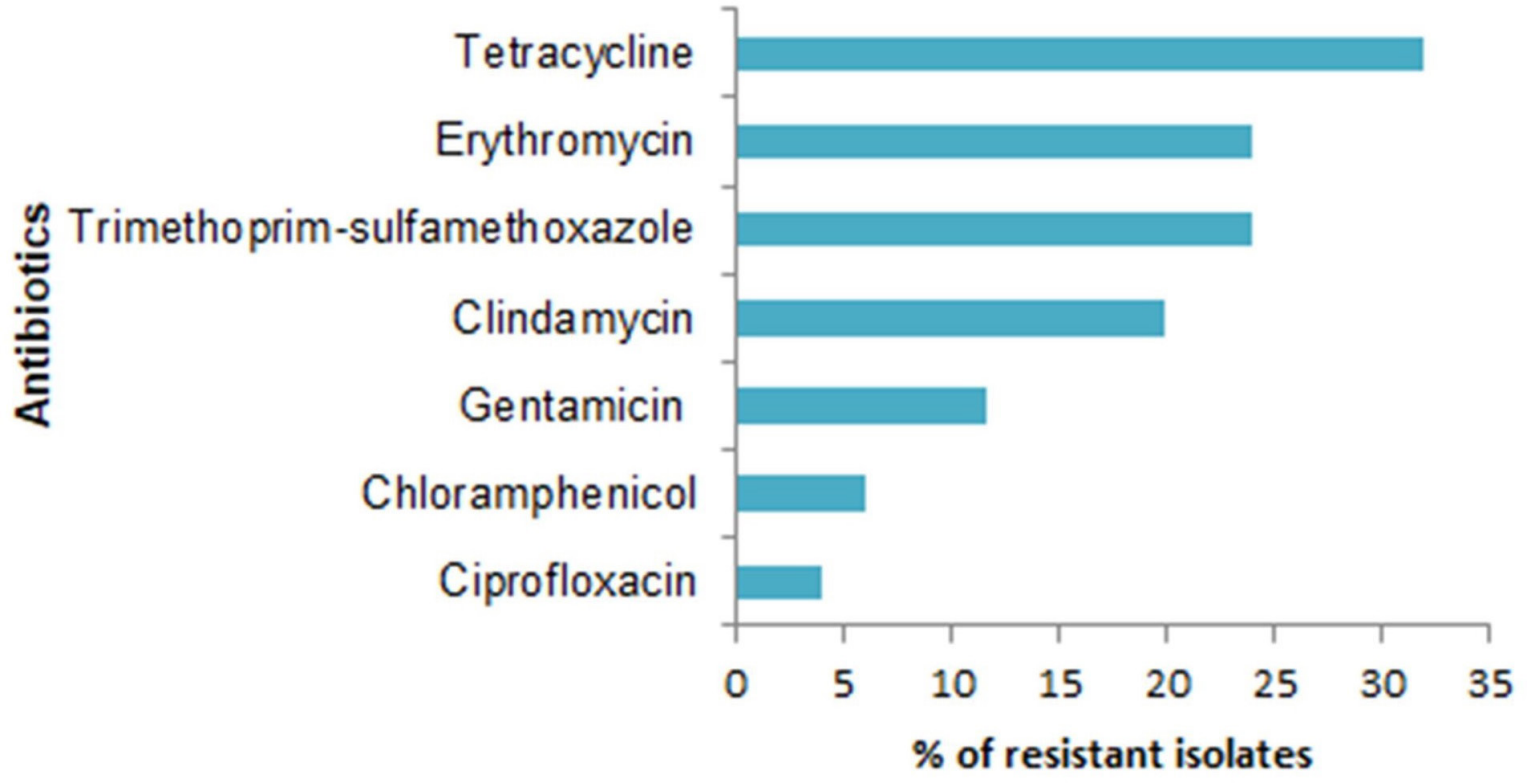
Antimicrobial susceptibility pattern of *S. aureus* isolates among HIV-infected children aged <15 years at ALERT hospital, Addis Ababa, Ethiopia.

Antimicrobial susceptibility patterns were determined for all the 50 *S. aureus* isolates. The highest rate of resistance was seen against tetracycline (32 %) followed by erythromycin (24 %) and trimethoprim-sulfamethoxazole (24 %) (Fig. 1).

Among all MRSA isolates the highest percentage of drug resistance was seen against tetracycline (35.2 %), erythromycin (29.4 %), clindamycin (29.4 %), and gentamicin (11.7 %), sulfamethoxazole-trimethoprim (5.9 %), chloramphenicol (5.9 %) and ciprofloxacin (5.9 %). The overall multidrug-resistance (MDR) rate in this study was 12 %.

## Discussion

Nasopharyngeal colonization and infection with *S. aureus* and MRSA is higher in HIV-infected children than those without HIV [22, 23]. Determining the *S. aureus* carriage rate and antibiotic resistance profiles is important in order to identify risk factors associated with *S. aureus* infection.

In the current study, we found that 5–7 years after introduction of PCV10 in Ethiopia, prevalence of *S. aureus* nasopharyngeal carriage was 27.3 %(50/183). Our result is in agreement with a similar study done in Northern Ethiopia 29 %(88/300) [19]. Similar prevalence has also been reported from South Africa, prior to the introduction of PCVs 25.6 %(91/355) [24]. Our finding was however lower than a report from results from a study in Brazil (45.16 %) before the introduction of PCVs [23]. This might be because of geographical differences, sample size, the use of antiretroviral drugs and the introduction of PCVs. Our results were however higher than *S. aureus* nasopharyngeal carriage prevalence among HIV-uninfected under-5 children reported from Uganda, 19.4 %(144/742) [25] and Ghana, 23.2 %(95/210) [26]. This might be due to the difference in the age group as most (70 %) of the children in the current study are aged 10–14 years.

Our finding indicates that the prevalence of MRSA nasopharyngeal colonization in HIV-infected children was 2.7 %(5/183). Our finding is similar to nasal MRSA colonization reported among children and adults in Northern Ethiopia 2.4 %(6/249) [27]. Our findings were however lower than the pharyngeal colonization reported in a similar group of HIV-infected children in Northern Ethiopia 9.7 %(29/300) [19]. In the study by Mulu *et al.* and colleagues, methicillin resistance was determined using cefoxitin discs whereas in the current study, we used *mecA* amplification to determine methicillin resistance. Although Cefoxitin resistance is known as a reliable surrogate marker for mecA-mediated methicillin resistance [28], there are studies that have questioned its accuracy [29]. Additional reasons for discrepancies in prevalence of MRSA could be differences in study locations and periods, differences in the implementation of infection prevention among the hospitals and rational antibiotic use practices in the two settings. In addition, in both studies, it is important to note that the reported prevalences are of the study cohorts and do not necessarily represent the overall situation in the country.

In this study, nasopharyngeal carriage of *S. pneumoniae* was 43.2 %(79/183). The results are higher than pharyngeal carriage reported among similar group of children in Northern Ethiopia 31(10.3 %) [27]. The study by Mulu and colleagues from Northern Ethiopia reports pharyngeal carriage and samples might not have been taken from the nasopharynx. Our results were however lower than those reported from the same hospital as the current study at a later time point (2018–2019), 52 %(26/50) [30]. The differences in carriage prevalence might be due to differences in the median ages of the study populations, which was the 53.5 months' range (24–89) in the study by Lemma and colleagues while in the current study the median age is the 132 months' range (36–168). Our results were also similar to those reported among HIV-uninfected children of a similar age range in Southern Ethiopia [31].

In multivariable analyses, there was an inverse association between *S. aureus* and *S *. *pneumoniae* nasopharyngeal colonization in this group of HIV-infected children 5 to 7 years after introduction of PCV10 in the country. Accordingly HIV-infected children who are colonized with *S. pneumoniae* are less likely to be colonized with *S. aureus *. A negative association between carriage of *S. aureus* and PCV7 vaccine type *S. pneumoniae* was first reported close to two decades ago by two studies from Israel [32] and the Netherlands [33] before the introduction of pneumococcal conjugate vaccines. Since then, other studies from different parts of the world have reported similar results [34, 35] mainly among healthy or HIV-uninfected children. However, studies among HIV-infected children from South Africa indicated lack of association between the nasopharyngeal colonization of *S. aureus* and *S. pneumoniae* [14, 24]. The possible reasons for the lack of competition between the two bacteria in HIV-uninfected children included reduced mucosal immunity and therefore decreased immunological pressure and increased exposure to respiratory pathogens [24]. Other authors however suggested that a secondary host-related mechanism associated mainly with CD4^+^ T cells might play an important role in the pathway of interaction between *S. pneumoniae* and *S. aureus* [36]. The studies from South Africa did not study the correlation between CD4^+^ T cell counts and bacterial colonization or interaction. In the current study, the median (IQR) CD4 +T cell count of the study participants was 732 (524-924) cells/mm^3^.

According to the Ethiopian national guideline for HIV prevention, care and treatment, for all children living with HIV, ART is given as early as possible regardless of their WHO clinical stages and CD4^+^ T cell counts/percentage [37]. Studies indicate that early HIV diagnosis and early ART reduce infant mortality and HIV progression [38]. In addition, in HIV-infected infants with early ART treatment CD4^+^ T cell counts stabilize at the highest levels possible [39]. We therefore hypothesize the inverse interaction between *S. aureus* and *S. pneumoniae* seen in the HIV-infected children in this study, similar to previous reports on the inverse interaction between *S. aureus* and *S. pneumoniae* in HIV-uninfected children, is due to the impact of ART treatment.

People living with HIV are at increased risk of acquisition and infection with drug-resistant bacteria [6]. In this study, among both MSSA and MRSA isolates, the highest level of resistance was seen against tetracycline, which was consistent with a similar study among HIV-infected children in Northern Ethiopia [40] although a higher percentage of resistance to tetracycline among MRSA isolates (72 %) compared to our study (35.2 %) were reported. The low rates of resistance to most of the antibiotics tested observed in this study might suggest that most of the MSSA and MRSA were community-acquired strains [41].

Our study had some limitations. Firstly, prevalence of *S. aureus* colonization might have been underestimated since we sampled only the nasopharynx. Since our results originate from only one hospital, the results might not be fully representative of children in the whole of Addis Ababa or Ethiopia. Because serotyping was not performed for the *S. pneumoniae* isolates, we could not determine the relationship between vaccine type and non-vaccine type *S. pneumoniae* colonization and *S. aureus* colonization. In addition, our focus was only on colonization and the study was not designed to identify disease caused by these pathogens.

## Conclusions

The results of this study suggest that 5 to 7 years after introduction of PCV10 in Ethiopia and more than 12 years after introduction of ART in Ethiopia, the nasopharyngeal colonization rate of *S. aureus* in HIV-infected children at ALERT hospital, Addis Ababa, Ethiopia was 27.3 and 10 % of the *S. aureus* isolates were MRSA. In addition, there was an inverse relationship between the colonization of *S. aureus* and *S. pneumoniae *.

## References

[1] Turner NA, Sharma-Kuinkel BK, Maskarinec SA, Eichenberger EM, Shah PP (2019). Methicillin-resistant *Staphylococcus aureus*: an overview of basic and clinical research. Nat Rev Microbiol.

[2] Wertheim HFL, Melles DC, Vos MC, van Leeuwen W, van Belkum A (2005). The role of nasal carriage in *Staphylococcus aureus* infections. Lancet Infect Dis.

[3] Peacock SJ, Justice A, Griffiths D, de Silva GDI, Kantzanou MN (2003). Determinants of acquisition and carriage of *Staphylococcus aureus* in infancy. J Clin Microbiol.

[4] Hu X, Hu K, Liu Y, Zeng L, Hu N (2022). Risk factors for methicillin-resistant *Staphylococcus aureus* colonization and infection in patients with human immunodeficiency virus infection: a systematic review and meta-analysis. J Int Med Res.

[5] Shet A, Mathema B, Mediavilla JR, Kishii K, Mehandru S (2009). Colonization and subsequent skin and soft tissue infection due to methicillin-resistant *Staphylococcus aureus* in a cohort of otherwise healthy adults infected with HIV type 1. J Infect Dis.

[6] Olaru ID, Tacconelli E, Yeung S, Ferrand RA, Stabler RA (2021). The association between antimicrobial resistance and HIV infection: a systematic review and meta-analysis. Clin Microbiol Infect.

[7] Safdar N, Bradley EA (2008). The risk of infection after nasal colonization with *Staphylococcus aureus*. Am J Med.

[8] Sakr A, Brégeon F, Mège J-L, Rolain J-M, Blin O (2018). *Staphylococcus aureus* nasal colonization: an update on mechanisms, epidemiology, risk factors, and subsequent infections. Front Microbiol.

[9] Neupane K, Rayamajhee B, Acharya J, Rijal N, Shrestha D (2018). Comparison of nasal colonization of methicillin-resistant *Staphylococcus aureus* in HIV-infected and non-HIV patients attending the national public health laboratory of central nepal. Can J Infect Dis Med Microbiol.

[10] Ferreira D de C, Silva GR da, Cavalcante FS, Carmo FL do, Fernandes LA (2014). Methicillin-resistant *Staphylococcus aureus* in HIV patients: risk factors associated with colonization and/or infection and methods for characterization of isolates - a systematic review. Clinics.

[11] Tacconelli E, Carrara E, Savoldi A, Harbarth S, Mendelson M (2018). Discovery, research, and development of new antibiotics: the WHO priority list of antibiotic-resistant bacteria and tuberculosis. Lancet Infect Dis.

[12] Broekema NM, Van TT, Monson TA, Marshall SA, Warshauer DM (2009). Comparison of cefoxitin and oxacillin disk diffusion methods for detection of mecA-mediated resistance in *Staphylococcus aureus* in a large-scale study. J Clin Microbiol.

[13] Akcam FZ, Tinaz GB, Kaya O, Tigli A, Ture E (2009). Evaluation of methicillin resistance by cefoxitin disk diffusion and PBP2a latex agglutination test in mecA-positive *Staphylococcus aureus*, and comparison of mecA with femA, femB, femX positivities. Microbiol Res.

[14] Madhi SA, Adrian P, Kuwanda L, Cutland C, Albrich WC (2007). Long-term effect of pneumococcal conjugate vaccine on nasopharyngeal colonization by *Streptococcus pneumoniae*--and associated interactions with *Staphylococcus aureus* and *Haemophilus influenzae* colonization--in HIV-infected and HIV-uninfected children. J Infect Dis.

[15] Dukers-Muijrers NHTM, Stobberingh E, Beisser P, Boesten RCH, Jacobs P (2013). Nasal carriage of *Streptococcus pneumoniae* serotypes and *Staphylococcus aureus* in *Streptococcus pneumoniae*-vaccinated and non-vaccinated young children. Epidemiol Infect.

[16] Veenhoven R, Bogaert D, Uiterwaal C, Brouwer C, Kiezebrink H (2003). Effect of conjugate pneumococcal vaccine followed by polysaccharide pneumococcal vaccine on recurrent acute otitis media: a randomised study. Lancet.

[17] Negash AA, Asrat D, Abebe W, Hailemariam T, Hailu T (2019). Bacteremic community-acquired pneumonia in Ethiopian children: etiology, antibiotic resistance, risk factors, and clinical outcome. Open Forum Infect Dis.

[18] Reiss-Mandel A, Regev-Yochay G (2016). *Staphylococcus aureus* and *Streptococcus pneumoniae* interaction and response to pneumococcal vaccination: Myth or reality?. Hum Vaccin Immunother.

[19] Mulu W, Yizengaw E, Alemu M, Mekonnen D, Hailu D (2018). Pharyngeal colonization and drug resistance profiles of *Morraxella catarrrhalis*, *Streptococcus pneumoniae*, *Staphylococcus aureus*, and *Haemophilus influenzae* among HIV infected children attending ART clinic of Felegehiwot referral hospital, Ethiopia. PLoS One.

[20] Yamagishi J, Sato Y, Shinozaki N, Ye B, Tsuboi A (2016). Comparison of boiling and robotics automation method in DNA extraction for metagenomic sequencing of human oral microbes. PLoS One.

[21] Pérez-Roth E, Claverie-Martín F, Villar J, Méndez-Alvarez S (2001). Multiplex PCR for simultaneous identification of *Staphylococcus aureus* and detection of methicillin and mupirocin resistance. J Clin Microbiol.

[22] Kenga DB, Gebretsadik T, Simbine S, Maússe FE, Charles P (2021). Community-acquired bacteremia among HIV-infected and HIV-exposed uninfected children hospitalized with fever in Mozambique. Int J Infect Dis.

[23] D’Avila NEM, Zhang L, Miller RG, D’Avila AC, Conceição APC (2008). High prevalence of nasopharyngeal colonization by *Staphylococcus aureus* among children with HIV-1 infection in extreme Southern Brazil. J Trop Pediatr.

[24] McNally LM, Jeena PM, Gajee K, Sturm AW, Tomkins AM (2006). Lack of association between the nasopharyngeal carriage of *Streptococcus pneumoniae* and *Staphylococcus aureus* in HIV-1-infected South African children. J Infect Dis.

[25] Kateete DP, Asiimwe BB, Mayanja R, Mujuni B, Bwanga F (2019). Nasopharyngeal carriage, spa types and antibiotic susceptibility profiles of *Staphylococcus aureus* from healthy children less than 5 years in Eastern Uganda. BMC Infect Dis.

[26] Dayie NTKD, Osei M-M, Opintan JA, Tetteh-Quarcoo PB, Kotey FCN (2021). Nasopharyngeal carriage and antimicrobial susceptibility profile of *Staphylococcus aureus* among children under five years in Accra. Pathogens.

[27] Gebremedhn G, Gebremariam TT, Wasihun AG, Dejene TA, Saravanan M (2016). Prevalence and risk factors of methicillin-resistant *Staphylococcus aureus* colonization among HIV patients in Mekelle, Northern Ethiopia. Springerplus.

[28] Swenson JM, Lonsway D, McAllister S, Thompson A, Jevitt L (2007). Detection of mecA-mediated resistance using reference and commercial testing methods in a collection of *Staphylococcus aureus* expressing borderline oxacillin MICs. Diagn Microbiol Infect Dis.

[29] Mimica MJ, Berezin EN, Carvalho RLB, Mimica IM, Mimica LMJ (2007). Detection of methicillin resistance in *Staphylococcus aureus* isolated from pediatric patients: is the cefoxitin disk diffusion test accurate enough?. Braz J Infect Dis.

[30] Lemma M, Bekele Y, Petkov S, Hägglund M, Petros B (2020). *Streptococcus pneumoniae* nasopharyngeal carriage among PCV-10-vaccinated HIV-1-infected children with maintained serological memory in Ethiopia. Pathogens.

[31] Wada FW, Tufa EG, Berheto TM, Solomon FB (2019). Nasopharyngeal carriage of *Streptococcus pneumoniae* and antimicrobial susceptibility pattern among school children in South Ethiopia: post-vaccination era. BMC Res Notes.

[32] Regev-Yochay G, Dagan R, Raz M, Carmeli Y, Shainberg B (2004). Association between carriage of *Streptococcus pneumoniae* and *Staphylococcus aureus* in Children. JAMA.

[33] Bogaert D, van Belkum A, Sluijter M, Luijendijk A, de Groot R (2004). Colonisation by *Streptococcus pneumoniae* and *Staphylococcus aureus* in healthy children. Lancet.

[34] Shiri T, Nunes MC, Adrian PV, Van Niekerk N, Klugman KP (2013). Interrelationship of *Streptococcus pneumoniae*, *Haemophilus influenzae* and *Staphylococcus aureus* colonization within and between pneumococcal-vaccine naïve mother-child dyads. BMC Infect Dis.

[35] Esposito S, Terranova L, Ruggiero L, Ascolese B, Montinaro V (2015). *Streptococcus pneumoniae* and *Staphylococcus aureus* carriage in healthy school-age children and adolescents. J Med Microbiol.

[36] Bogaert D, Nouwen J, Hermans PWM, Belkum A van (2006). Lack of Interference between *Streptococcus pneumoniae* and *Staphylococcus aureus* in HIV-infected individuals?. J Infect Dis.

[37] FMOH (2014). National Guidelines for Comprehensive HIV Prevention, Care and Treatment.

[38] Violari A, Cotton MF, Gibb DM, Babiker AG, Steyn J (2008). Early antiretroviral therapy and mortality among HIV-infected infants. N Engl J Med.

[39] Lewis J, Payne H, Walker AS, Otwombe K, Gibb DM (2017). Thymic output and CD4 T-cell reconstitution in HIV-infected children on early and interrupted antiretroviral treatment: evidence from the children with HIV early antiretroviral therapy trial. Front Immunol.

[40] Lemma MT, Zenebe Y, Tulu B, Mekonnen D, Mekonnen Z (2015). Methicillin resistant *Staphylococcus aureus* among HIV infected pediatric patients in Northwest Ethiopia: carriage rates and antibiotic co-resistance profiles. PLoS One.

[41] Dietrich DW, Auld DB, Mermel LA (2004). Community-acquired methicillin-resistant *Staphylococcus aureus* in southern New England children. Pediatrics.

